# Does the ingestion of a 24 hour low glycaemic index Asian mixed meal diet improve glycaemic response and promote fat oxidation? A controlled, randomized cross-over study

**DOI:** 10.1186/s12937-017-0258-1

**Published:** 2017-07-12

**Authors:** Stefan Gerardus Camps, Bhupinder Kaur, Rina Yu Chin Quek, Christiani Jeyakumar Henry

**Affiliations:** 1 Clinical Nutrition Research Centre (CNRC), Singapore Institute for Clinical Sciences (SICS), Agency for Science, Technology and Research (A*STAR) and National University Health System, Centre for Translational Medicine, Yong Loo Lin School of Medicine, 14 Medical Drive #07-02, MD 6 Building, Singapore, 117599 Singapore; 2 Department of Biochemistry, Yong Loo Lin School of Medicine, National University of Singapore, S14 Level 5, Science Drive 2, Singapore, 117543 Singapore

**Keywords:** Glycaemic index, Mixed meals, 24 h diet, Glycaemic response, Continuous glucose monitoring, Substrate oxidation, Indirect calorimetry, Whole body calorimeter

## Abstract

**Background:**

The health benefits of consuming a low glycaemic index (GI) diet to reduce the risk of type 2 Diabetes are well recognized. In recent years the GI values of various foods have been determined. Their efficacy in constructing and consuming a low GI diet over 24 h in modulating glycaemic response has not been fully documented. The translation of using single-point GI values of foods to develop a 24 h mixed meal diet can provide valuable information to consumers, researchers and dietitians to optimize food choice for glycaemic control. By using GI values of foods to develop mixed meals, our study is the first to determine how both blood glucose and substrate oxidation may be modulated over 24 h.

**Methods:**

The study included 11 Asian men with a BMI between 17–24 kg/m^2^ who followed both a 1-day low GI and 1-day high GI diet in a randomized, controlled cross-over design. Test meals included breakfast, lunch, snack and dinner. Glycaemic response was measured continuously for over 24 h and postprandial substrate oxidation for 10 h inside a whole body calorimeter.

**Results:**

The low GI diet resulted in lower 24 h glucose iAUC (860 ± 440 vs 1329 ± 614 mmol/L.min; *p* = 0.014) with lower postprandial glucose iAUC after breakfast (p < 0.001), lunch (*p* = 0.009), snack (*p* = 0.012) and dinner (*p* = 0.003). Moreover, 24 h mean amplitude of glycaemic excursion was lower during the low GI vs high GI diet (1.44 ± 0.63 vs 2.33 ± 0.82 mmol/L; *p* < 0.001). Simultaneously, decrease in 10 h fat oxidation was less during the low vs high GI diet (−0.033 ± 0.021 vs −0.050 ± 0.017 g/min; p < 0.001), specifically after breakfast (*p* < 0.001) and lunch (*p* < 0.001).

**Conclusions:**

Our study corroborates that using low GI local foods to construct a 24 h low GI diet, is able to reduce glycaemic response and variability as recorded by continuous glucose monitoring. Our observations also confirm that a low GI diet promotes fat oxidation over carbohydrate oxidation when compared to a high GI diet. These observations provide public health support for the encouragement of healthier nutrition choices by consuming low GI foods.

**Trial registration:**

NCT 02631083 (Clinicaltrials.gov).

## Background

Glycaemic index (GI) of foods is a method of classifying foods based on postprandial glycaemic responses [1]. This response has been quantified and there is a large body of published GI values of food and food products available [2, 3]. Despite numerous papers extolling the health benefits of consuming a low GI foods to reduce the risk of type 2 Diabetes and metabolic syndrome [4], a major criticism of its utility has been that many studies have only reported the effects of feeding individual low GI foods, notably as incremental area under curve of blood glucose over a 2 h period. Little is known about the effect of feeding a low or high GI diet at breakfast, lunch, snack and dinner on 24 h glucose profiles in young healthy Asians. Findings from continuous glucose monitoring in Caucasians with Diabetes indicate that consumption of a low GI diet may reduce glucose excursions and improve glycaemic control [5–7]. It is imperative to investigate the 24 h glucose response to feeding low or high GI mixed meal diets over a 24 h period in the Asian population, if people are to be encouraged to follow a low GI diet over several days.

Although the concept of GI has been widely accepted in Canada, parts of Europe and Australia, it still remains a novelty in Asia. This is paradoxical as the impact and importance of consuming a low GI diet may be of greater significance in Asia since the Asian phenotype has been shown to be more susceptible to Diabetes compared to Caucasians [8, 9]. Additionally, the Asian diets are predominantly based on a high carbohydrate, high GI foods including rice. Indeed, in certain regions of Asia, example Myanmar, over 70% of the calories consumed comes from the consumption of rice [10, 11]. In contrast to several studies and reports of GI values of Western foods, it is only in recent years that the GI of various Asian foods has been determined [12–16].

With the lack of published GI values of local foods, Henry and colleagues among others conducted several studies which determined the GI values of local commonly consumed foods, that were not previously tested [13]. The GI values of local foods provide valuable information to consumers, researchers and dietitians to optimize food choice for glycaemic control. However, these GI values have been single food measurements conducted in an Asian population and need to be translated to mixed meals typically consumed in Singapore. Using GI values obtained from local foods, our study is the first to determine how 24 h blood glucose may be modulated in healthy Asians.

In addition to glycaemic health improvement, there is an increasing interest in the use of low GI foods in the management and prevention of obesity. Low GI foods have been hypothesized to benefit weight control by promoting satiety and fat oxidation at the expense of carbohydrate oxidation [17–19]. A recent published study by our group showed that consumption of low GI foods favoured an increase in fat oxidation in Asian subjects [20]. It is necessary to take traditional eating habits of the Asian population into account to allow a better selection of local foods with beneficial effects to minimize hyperglycaemia and increase fat oxidation.

Using continuous glucose monitoring and indirect calorimetry, our goal was to determine simultaneously, the glycaemic response and effect on substrate oxidation of a low GI mixed meal sequence developed with local Asian foods. The novelty of this study was that instead of single food GI studies, we investigated in an acute design, if consuming mixed meals based on local foods that are low in GI can reduce glycaemia over 24 h and increase fat oxidation. As the mixed meals were based on foods typically consumed in South-East Asia, this information can be relevant not only to plan diabetes diets but also to reduce the risk of developing type 2 Diabetes and obesity in this region. This study was designed to use commonly consumed foods sold mostly in coffee shops and hawker centres in Singapore that represent the type of foods commonly consumed all over Asia.

## Methods

This randomized control trial was conducted at the Clinical Nutrition Research Centre (CNRC) within the Singapore Institute of Clinical Sciences (SICS), Agency of Science Technology and Research (A*STAR), Singapore. The study received ethical approval from the Domain Specific Review Board of the National Healthcare Group in Singapore (NHG DSRB Reference No. 2014/00960). Written informed consent was obtained from all eligible participants before commencement and the research procedures and trial protocols were followed in accordance to good clinical practice (GCP) guidelines and with the ethical standards in concordance to the Declaration of Helsinki, 1983. This trial was registered within clinicaltrials.gov under trial registration no. NCT 02631083.

Eleven healthy Chinese adults were recruited using a variety of methods which included flyers, online advertisements and personal communication. Subjects underwent an initial screening and measurements included anthropometry (height, weight, waist and hip circumference), fat percentage via air displacement plethysmography (BodPod, Life Measurements Inc, Concord, CA, USA), blood pressure, resting heart rate, fasting blood glucose and %HbA1C. Physical activity was quantified using the questionnaire by Baecke et al. [21]. Eating behaviour was quantified using a Dutch eating behaviour questionnaire by Van Strien et al. [22]. Eleven subjects fulfilled the following inclusion criteria: male, age: 21–40 years; body mass index 17–25 kg/m^2^; no metabolic diseases; not on prescription medication; not allergic/intolerant to any of the test foods; not intentionally restricting food intake; fasting blood glucose <6 mmol/L. Baseline anthropometric and biochemistry data of the study participants are shown in Table 1.

**Table 1:** Baseline measurements of study participants (*n* = 11)

Characteristic	Mean ± SD
Age (years)	24.5 ± 2.0
Height (m)	1.75 ± 0.1
Weight (kg)	67.0 ± 4.7
BMI (kg/m^2^)	21.9 ± 1.4
Waist circumference (cm)	74.7 ± 4.5
Fasting blood glucose (mmol/L)	4.6 ± 0.4
HbA1c (%)	5.1 ± 0.2
Body fat (%)	16.0 ± 2.8
Basal metabolic rate	1456 ± 97
Systolic blood pressure (mmHg)	115.7 ± 9.7
Diastolic blood pressure (mmHg)	69.5 ± 9.2

### Study design

**Fig. 1:**
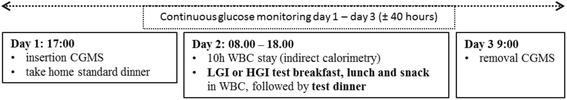
Study protocol. On day 1, the continuous glucose monitoring system (CGMS) was inserted in the afternoon. Subjects consumed a standardized dinner followed by an overnight fast. On day 2, subjects entered the whole body calorimeter (WBC) in a fasted state at 08:00, where basal metabolic rate (BMR) was measured for 45 min. Breakfast, lunch and snack low glycaemic index (LGI) or high glycaemic index (HGI) test meals were provided in the calorimeter. Subjects remained sedentary during the 10 h in the calorimeter. Subjects were allowed to leave the calorimeter at 18:00, after which the test dinner was provided. The CGMS was removed on day 3

The study had a randomized, controlled cross-over design with subjects attending two test sessions separated by a wash-out period of at least five days. The two test session included either a low glycaemic index (LGI) or a high glycaemic index (HGI) meal sequence (breakfast, lunch, snack and dinner). Participants were advised not to perform any rigorous activities three days prior to the study and during the study session. Each test session spanned over three consecutive days from 17:00 on Day 1 till 9:00 on Day 3 consisting of over 42 h continuous glucose monitoring (CGM) and a 10 h measurement of energy expenditure and substrate oxidation in a whole body calorimeter (WBC). During Day 2, participants stayed in the WBC room from 08:00 to 18:00 (10 h). For the first 45 min, they were asked to lie in a supine position on the bed to measure their basal metabolic rate (BMR). Participants were then given the LGI or HGI test breakfast, lunch and snack to consume. After the stay in the WBC, a LGI or HGI dinner was provided. Participants were free to study, surf the net, watch television, listen to radio, use the telephone or lie on the bed however, they were not allowed to sleep during their time in the WBC. They were also encouraged to keep to one activity after consuming the meal and to minimize movement. A schematic study flow is presented in Fig. 1. Online computer software was used for simple randomization of the sequence of the treatment diets (www.randomizer.org).

### Test meals

A standardized dinner was provided on Day 1 consisting of a ready-to-eat meal of teriyaki chicken with rice, one drink and one jelly (energy: 879 kcal; Protein: 44.3 g; Fat: 18.3 g; Carbohydrate: 132.7 g).

Locally consumed foods were selected to construct the high and low GI test meals:

- *Low GI breakfast*: Milo (Nestle, Singapore) GI: 36, low fat milk (Paul’s, Australia) GI: 37, lemon puff biscuits (Khong Guan, Singapore) GI:49; *High GI breakfast*: pink rice cake (steamed sticky rice cake with dried pork radish filling; also called *poon kueh*) GI:97 (purchased from a local hawker stall, Singapore), rice cracker (Bin-Bin, Singapore) GI:83, Nestum cereal drink made with water (Nestle, Malaysia) GI: 77.
- *Low GI lunch*: bee hoon (thin rice vermicelli) (Fairprice, Singapore) GI: 33, teriyaki chicken (Charoen Pokphand Intertrade, Singapore), fresh spinach GI:15; *High GI lunch*: mee pok (flat, yellow egg noodle varying in thickness and width) (Fortune, Singapore) GI: 73, fresh carrots GI: 49, teriyaki chicken (Charoen Pokphand Intertrade, Singapore), rice cracker (Bin-Bin, Singapore) GI:83. Both noodles for LGI and HGI were flavoured with some ketchup, sesame oil, dark soy sauce, water.
- *Low GI snack:* kaya butter toast (toasted bread with butter and coconut jam made from coconut milk, eggs and sugar) (purchased from a local hawker stall, Singapore) GI:49, Milo (Nestle, Singapore) GI: 36, low fat milk (Paul’s, Australia) GI: 37; *High GI snack*: steamed glutinous rice with chicken (steamed sticky rice with chicken and mushroom, also called *lo mai gai*) (purchased from a local hawker stall, Singapore) GI: 106, canned ice lemon tea (Fraser and Neave, Malaysia) GI: 74
- *Low GI dinner:* parboiled basmati rice (Diabetic Specialities Pte Ltd, Singapore) GI: 55, chicken stock (Knorr chicken stock, Malaysia), fresh spinach, teriyaki chicken (Charoen Pokphand Intertrade, Singapore); *High GI dinner:* glutinous rice (sticky rice, it has a low amylose content and is especially sticky when cooked) (New Moon, Tek Seng Rice Mill Co. Ltd, Thailand) GI:92, chicken stock (Knorr chicken stock, Malaysia), fresh carrots GI: 49, margarine spread (Flora, Unilever, Australia), teriyaki chicken (Charoen Pokphand Intertrade, Singapore), rice cracker (Bin-Bin, Singapore) GI:83. The energy values and macronutrient composition of the test meals are provided in Tables 2 and 3. The daily meals provided to the subjects matched their daily energy requirements based on the measured basal metabolic rate of a subject multiplied by a physical activity level of 1.5.

**Table 2:** Macronutrient composition and GI values of the test diets provided in the study

	Low GI diet	High GI diet
Energy % Carbohydrate	62	64
Energy % Fat	23	23
Energy % Protein	14	12
Meal GI breakfast	43	90
Meal GI lunch	32	73
Meal GI snack	43	95
Meal GI dinner	53	85

GI: glycaemic index

**Table 3:** Foods used to construct the test meals provided in the study

Low GI diet	High GI diet
Breakfast	Breakfast
Milo, milk (low fat) lemon puff biscuits	poon kueh (pink rice cake), rice cracker Nestum cereal drink (made with water)
Lunch	Lunch
bee hoon (rice vermicelli), sauce (ketchup, sesame oil, dark soy, water), teriyaki chicken, spinach (boiled)	mee pok (flat egg noodle), sauce (ketchup, sesame oil, dark soy, water), teriyaki chicken, carrots (boiled), rice cracker
Snack	Snack
kaya butter toast (kaya is made from coconut milk, eggs and sugar), Milo, milk (low fat)	steamed glutinous rice with chicken (lo mai gai), ice lemon tea
Dinner	Dinner
parboiled basmati rice, chicken stock, teriyaki chicken, spinach (boiled)	glutinous rice, chicken stock teriyaki chicken, carrots (boiled then mixed with margarine), rice cracker

*GI*, glycaemic index

The low and high GI meals were designed to obtain as wide a difference in the calculated meal GI values. The GI of individual foods were obtained using GI values from recognized tables [23] and from manufacturers’ information. The meal GI was calculated using the mixed meal formula [24]. Subjects were requested to consume both breakfast and snack within 15 min and lunch within 20 min.

### Interstitial glucose measurement

Continuous glucose monitoring (CGM) (iPro™2 Professional CGM-Medtronic MiniMed, Northbridge, CA, USA) was used to measure glycaemic response, defined as the primary outcome. The insertion was performed on Day 1 at 17:00 and the sensor was removed on Day 3 of the study at 9:00. Data was collated and processed using online software (Medtronic Diabetes CareLink iPro; carelink.minimed.eu). The data reported in this paper represent 24 h interstitial glucose readings recorded every 5 min from 6:00 on Day 2 to 6:00 on Day 3. During each test session, the CGM sensor was calibrated against finger-stick blood glucose measurements four times a day before every meal and before sleeping using the OneTouch®Ultra®2 blood glucose meter (LifeScan, Inc., Milpitas, CA, USA). A cross-over design with a minimum of 8 subjects would be sufficient to detect a 15% change in area under the glucose curve (24 h) with a power of 0.85 at a significance level of 0.05 as adapted from Brynes et al. [25, 26].

### Energy expenditure and substrate oxidation

Basal metabolic rate (BMR), respiratory quotient (RQ) and substrate oxidation were measured for 10 h using a dual room WBC facility based on the system described by Schoffelen et al. [27]. Each WBC room is an open circuit, airtight indirect calorimeter with a total volume of 13.5 m^3^, furnished with a single-bed, a foldable chair, a bureau with built-in sink, deep-freeze toilet (Special Product, Mulders), a color television, an alarm clock, a radio, a telephone, a laptop, WIFI connection and an automated intercom for communication between the researcher and the participant. It is built to mimic a normal room with two windows for visual contact between the researcher and participant.

During test sessions, gaseous exchanges were measured continuously as oxygen consumption and carbon dioxide production through differences between inlet and outlet oxygen (O_2_) and carbon dioxide (CO_2_) concentrations. Oxygen concentration was measured using a paramagnetic O_2_ analyser (Model AO2020, module Magnos206, ABB Automation GmbH, Germany) while carbon dioxide concentration was measured using an infrared photometer (Model AO2020, module Uras26, ABB Automation GmbH, Germany). The air samples are measured in an automated sequence and alternated with calibration span gas (18% O_2_, 0.8% CO_2_, and balance nitrogen) and zero (100% nitrogen) gases [27]. Gaseous exchanges were measured under standard temperature, pressure, and dry (STPD). The accuracy of the WBC chambers was routinely tested through the combustion of a known amount of methanol. The accuracy of O_2_ and CO_2_ measurements in our WBC facility were: O_2_ = 100.6 ± 0.5% (chamber 1) and 100.9 ± 0.4% (chamber 2), and CO_2_ = 99.2 ± 0.5% (chamber 1) and 99.7 ± 0.5% (chamber 2), while the coefficient of variation was 3.0% (*n* = 21) for repeated 30-min RMR measurements with our WBC facility.

BMR was calculated based on volume of O_2_ consumption (VO_2_) and CO_2_ production (VCO_2_) using the Weir equation [28]. Postprandial substrate oxidation and respiratory quotient (RQ) were calculated from urinary nitrogen excretion, oxygen consumption and carbon dioxide production [29]. Urine samples were collected in the WBC over 10 h in a 3 litre urine collection container (Unisafe®, Canada). The total volume of urine over 10 h was measured and a homogenized urine sample was stored for nitrogen analysis. Nitrogen content (%) was measured using the copper catalyst Kjeldahl method (AOAC Official Method 984.13). Protein oxidation (g/min) was calculated by multiplying 10 h urinary nitrogen (g) by 6.25 and converted to per minute values. CHO oxidation and fat oxidation were calculated by using the following equations based on the volumes of O_2_ consumed and CO_2_ produced in oxidation of glucose, fat and protein as published by Frayn [30]: CHO oxidation (g/min) = −3.21 × O_2_ (l/min) + 4.55 × CO_2_ (l/min) - 2.87 × N (g/min) and fat oxidation (g/min) = 1.67 × O_2_ (l/min) - 1.67 × CO_2_ (l/min) – 1.92 × N (g/min).

### Statistical analysis

All statistical analyses were performed using Statistical Package for the Social Sciences version 23 (SPSS Inc.). Data and figures were processed in a Microsoft Excel spreadsheet (Microsoft Corporation). Values were presented as mean ± SD unless otherwise stated. Prior to statistical analysis, the normality of the data was assured using the Shapiro-Wilks test.

The primary outcome of this study was to determine how a 24 h meal sequence including LGI and HGI local foods impacts on 24 h glycaemic response and variability and substrate oxidation.

First, the baseline glucose value for each subject was determined from the 1 h average CGM interstitial glucose readings in a fasted state from 6:00 to 7:00 on day 2 and used to calculate the change in glucose level for all 40 h. Glycaemic response was expressed as the incremental area under the curve (iAUC) and calculated using the trapezoidal rule and the change in glucose above baseline [31, 32]. The GR values were important for further analyses such as the GR iAUC calculations, CGMS glucose curve construction and statistics.

Second, mean amplitude of glycaemic excursion (MAGE) was assessed as an indicator for glycaemic variability during the day [33–35]. MAGE was calculated using EasyGV software (available free at www.easygv.co.uk), with this software being extensively reviewed [36].

Third, baseline substrate oxidation and RQ were calculated from 45 min steady-state measurement of BMR in a fasted state and used to assess 10 h and postprandial changes from baseline. Additionally, substrate oxidation was also expressed as the grams of carbohydrates, fat and protein oxidised. Glycaemic parameters are based on all 11 subjects with complete CGM data sets for both the LGI and HGI diets. Substrate oxidation and RQ results are based on 9 subjects with a complete set of whole body calorimeter and urine nitrogen data for both the LGI and HGI diets. During two test sessions, two subjects failed to produce an adequate and representative amount of urine for nitrogen analysis and these were excluded from the analysis for substrate oxidation. Paired t-test was performed to test the differences in the glycaemic response (one-tailed), MAGE and substrate oxidation (two-tailed) between LGI and HGI treatments diets and alpha (α) was set at 0.05 for statistical analyses.

## Results

The baseline characteristics of the subjects are given in Table 1. The anthropometric data were within normal ranges for this population.

### Continuous glucose monitoring interstitial glucose data

**Fig. 2:**
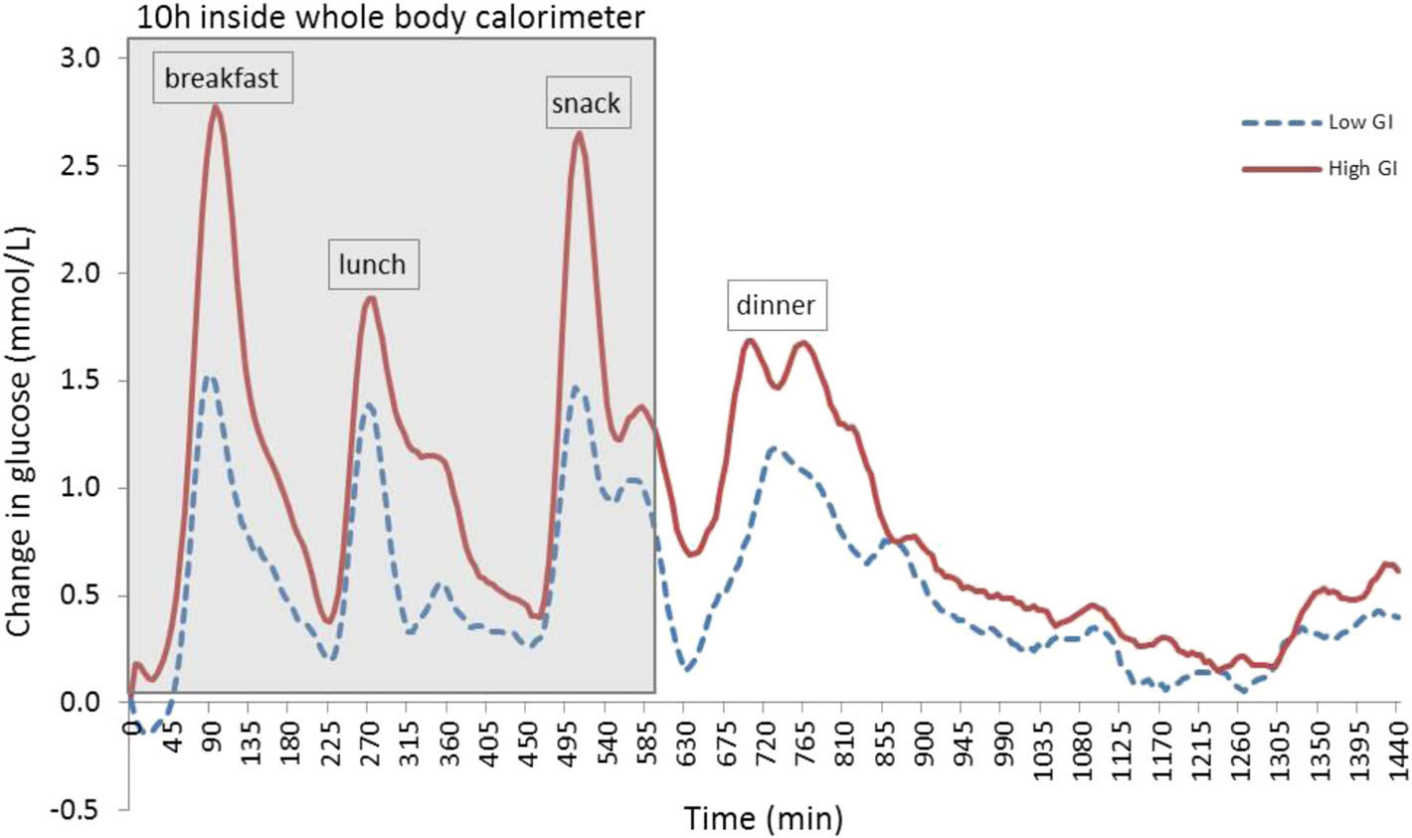
24 h average change in interstitial glucose concentrations from baseline from subjects on a low GI or high GI diet (*n* = 11). CGM, continuous glucose monitoring (Overnight fast range from 960 min to 1440 min)

The glycaemic profiles for the LGI and HGI diets are graphically presented in Fig. 2. The glycaemic outcome parameters, iAUC and MAGE, are presented in Table 4. Generally, the HGI intervention produced a sustained higher glycaemic response throughout the day (Fig. 2). During the LGI intervention, the iAUC following all meals (i.e. breakfast, lunch, snack and dinner) were significantly lower (*P* < 0.05) compared to the HGI intervention. Overnight iAUC following the test dinner was not significantly different after the LGI diet (*P* = 0.140). Over 24 h, there was a trend for a significantly lower iAUC during the LGI intervention (*P* = 0.056). During the 10 h in the WBC, there was a significantly lower GR during the LGI treatment compared to HGI (*P* = 0.012). Total daily AUC, which uses the absolute glucose values, was not significantly different between both treatments (*P* = 0.478). Glycaemic variability over the 24 h period, as assessed by MAGE showed a significantly lower variability during the LGI treatment (*P* = 0.003).

**Table 4:** Glycaemic outcome parameters for subjects on a low GI and high GI diet (*n* = 11)

Outcome measure	Low GI	High GI	*p*-value
24 h iAUC (mmol/L.min)	859.8 ± 439.7	1329.4 ± 613.6	0.014
iAUC in calorimeter (mmol/L.min) (10 h)	423.9 ± 234.3	706.0 ± 268.5	0.002
Breakfast iAUC (mmol/L.min)	115.4 ± 63.6	209.7 ± 62.6	<0.001
Lunch iAUC (mmol/L.min)	97.3 ± 61.0	162.1 ± 75.0	0.009
Snack iAUC (mmol/L.min)	129.2 ± 67.8	198.8 ± 84.4	0.012
Dinner iAUC (mmol/L.min)	122.7 ± 79.9	196.1 ± 127.6	0.003
Overnight iAUC (mmol/L.min) (after test dinner)	201.2 ± 148.9	259.0 ± 202.1	0.232
MAGE over 24 h (mmol/L)	1.44 ± 0.63	2.33 ± 0.82	<0.001

Values are expressed as mean ± SD; *GI*: glycaemic index; *MAGE*: mean amplitude of glycaemic excursion

*P*-values from paired students *t*-test when comparing glycaemic parameters between the low and high GI diet

During the 10 h in the calorimeter, absolute RQ was lower during the LGI diet (*p* = 0.018) and RQ increased more during the HGI diet (*P* < 0.001). The greater increase in RQ during the HGI diet was seen specifically after breakfast and lunch (*p* < 0.001) but not after the snack. Correspondingly, carbohydrate oxidation was lower during the LGI diet over the 10 h in the whole body calorimeter (*p* = 0.011) and increased more during the HGI diet measured over 10 h (*p* < 0.001), after breakfast (*p* < 0.001) and after lunch (*p* = 0.002) but not after the snack. 10 h fat oxidation was higher greater during the LGI diet (*p* = 0.002) and decreased less over 10 h (*p* < 0.001) and postprandially after breakfast (*p* < 0.001), lunch (*p* < 0.001) during the LGI diet compared to the HGI diet; there was a trend for a smaller decrease in fat oxidation after the LGI snack (*p* = 0.076) (Table 5).

**Table 5:** Substrate oxidation parameters for subjects on a low GI and high GI diet (*n* = 9), with incremental changes from pre-breakfast baseline

Outcome measure	Low GI	High GI	*p*-value
RQ (10 h)	0.887 ± 0.038	0.898 ± 0.032	0.018
Incremental RQ (10 h)	0.074 ± 0.038	0.099 ± 0.032	<0.001
Incremental RQ breakfast	0.064 ± 0.034	0.087 ± 0.026	<0.001
Incremental RQ lunch	0.071 ± 0.025	0.103 ± 0.026	<0.001
Incremental RQ snack	0.102 ± 0.046	0.115 ± 0.031	0.287
CHO oxidation (10 h) (g/min)	0.213 ± 0.044	0.229 ± 0.042	0.011
Incremental CHO oxidation (10 h) (g/min)	0.105 ± 0.044	0.132 ± 0.042	<0.001
Incremental CHO oxidation post-breakfast (g/min)	0.085 ± 0.039	0.106 ± 0.035	0.002
Incremental CHO oxidation post-lunch (g/min)	0.105 ± 0.026	0.140 ± 0.037	<0.001
Incremental CHO oxidation post-snack (g/min)	0.145 ± 0.051	0.161 ± 0.043	0.241
Fat oxidation (10 h) (g/min)	0.043 ± 0.021	0.034 ± 0.017	0.002
Incremental fat oxidation (10 h) (g/min)	−0.033 ± 0.021	−0.050 ± 0.017	<0.001
Incremental fat oxidation post-breakfast (g/min)	−0.032 ± 0.017	−0.047 ± 0.023	<0.001
Incremental fat oxidation post-lunch (g/min)	−0.033 ± 0.014	−0.052 ± 0.015	<0.001
Incremental fat oxidation post-snack (g/min)	−0.048 ± 0.025	−0.059 ± 0.018	0.076

Values are expressed as mean ± SD; *GI*: glycaemic index; *RQ* = respiratory quotient, *CHO* = carbohydrate

*P*-values from paired students *t*-test when comparing substrate oxidation between the low and high GI diet

Table 6 shows the grams of oxidized macronutrients for 10 h on a LGI and HGI diet and separately for breakfast (until lunch), lunch (until snack) and the snack (until end). During the LGI diet, significantly less carbohydrate (107.5 g vs 117.3 g, *p* = 0.002) and more fat (22.6 vs 17.3, *p* = 0.004) were oxidized compared to the HGI diet. Greater fat oxidation was observed during the low GI diet after breakfast (*p* = 0.042) and lunch (*p* < 0.001) with a similar trend after the snack (*p* = 0.079). There was no difference in protein oxidation between a LGI and HGI diet. There was no difference in energy expenditure over 10 h in the whole body calorimeter between the two dietary conditions (LGI = 749 kcal and HGI = 760 kcal), while there was a significant correlation between energy expenditure during LGI and HGI (R^2^ = 0.82, *p* < 0.001).

**Table 6:** Post-prandial oxidation of carbohydrates, fat and protein in grams for subjects on a low GI and high GI diet (*n* = 9)

	Low GI			High GI		
	CHO (g)	Fat (g)	Protein (g)	CHO (g)	Fat (g)	Protein (g)
Breakfast	27.1 (2.8)	6.3 (1.6)	8.0 (3.1)	28.5 (3.3)	5.3 (1.8)*	9.3 (1.1)
Lunch	50.2 (6.4)	11.3 (2.7)	13.7 (5.3)	56.4 (4.9)***	8.1 (2.1)**	15.9 (1.9)
Snack	30.2 (2.9)	4.9 (1.3)	7.4 (2.9)	32.4 (3.4)	3.9 (1.6)	8.6 (1.0)
Total	107.5 (8.7)	22.6 (4.9)	29.1 (11.3)	117.3 (9.8)**	17.3 (4.9)**	33.8 (4.1)

Values are expressed as mean ± SD; *GI*: glycaemic index; *CHO*: carbohydrate

*p <0.05, **p < 0.01, ***p < 0.001 (paired students *t*-test) when comparing the macronutrients between the low and high GI diet

## Discussion

The present study investigated the acute 24 h effects of the consumption of low GI mixed meals on simultaneously the blood glucose profile and substrate oxidation. It is the first study to corroborate that by constructing a 24 h low GI diet using local foods described as low GI, that these low GI Asian foods are capable of reducing 24 h glycaemic response and glycaemic variability and simultaneously promote fat oxidation over carbohydrate oxidation. The study expanded on previous work from our group on glycaemic index of single Asian foods on glucose response [13] and now included the measurement of substrate oxidation using a whole body calorimeter. The results are in line with the hypothesis that high GI foods result in hyperinsulinemia which in turn leads to less fat oxidation and possibly increased fat deposition [17, 37].

Previously, studies have shown that low GI foods can reduce the post-prandial glucose response compared to high GI foods and result in lower maximal and more stable glucose levels [4, 20, 38]. Foods classified lower in GI are more slowly digested and absorbed which result in a slower rate of appearance in the systemic circulation. This is in line with our results which show that a day of consumption of low GI mixed meals can lower 24 h glucose response. It is unique that this response is consistent over 24 h and is mediated by the consumption of commonly available low GI foods in Asia. Additionally, the results show a reduced glycaemic response after each low GI test meal with the greatest reduction occurring after breakfast, which could be explained by greater insulin sensitivity in the morning compared to later on the day [39]. There is good evidence to suggest that higher glycaemic variability can trigger more oxidative stress and it is considered a risk factor in the onset for type 2 Diabetes [23, 40]. Glycaemic variability, as assessed by mean amplitude of glycaemic excursion, showed a significantly lower variability over 24 h when on the low GI diet and is of specific clinical relevance for Diabetes prevention [23, 40]. In line with and in addition to current literature, locally consumed low GI mixed meals were able to down regulate the 24 h glycaemic response and variability in healthy Asian subjects over the day.

An additional beneficial effects of the consumption of low GI mixed meals was the consistent higher fat oxidation compared to high GI meals. During the 10 h in the whole body calorimeter, fat oxidation was higher when consuming low GI meals and this result was specifically apparent after breakfast and lunch. Not surprisingly, higher carbohydrate oxidation was seen during the high GI diet. These results are in line with previous literature, as studies showed increased fat oxidation and lower blood glucose during exercise following low GI meals [41–46]. Now, the results show that in a sedentary state, the low GI mixed meals are able to improve fat oxidation while at the same time improve glycaemic response and variability. It must be noted that these results are from healthy normal weight subjects, as Diaz and colleagues were unable to reproduce the ability to modify substrate partitioning in sedentary obese subjects [47]. They concluded that the lack of effect of serum insulin on fat oxidation may be due to the short time in which insulin concentrations are maintained at higher levels following high GI versus low GI meals. Obese people might be less susceptible for increased fat oxidation and these subjects were overfed with a high GI carbohydrate load irrespective of whether the meals were low or high.

The simultaneous measurement of substrate oxidation for 10 h during continuous glucose monitoring provided us with a unique perspective on both glucose flux and fat tissue accretion. The results show that following low GI meals, there is a reduced glucose response and increased fat oxidation compared to high GI. Previously, several authors have reported reduced fat deposition when fed a low GI diet [37]. Our results, implicate that the reduced fat deposition during a low GI diet is driven by increased fat oxidation. It has also been shown that after a high GI meal, the higher insulin to glucagon ratio as a result of the higher increase in insulin and inhibition of glucagon, increases the uptake of carbohydrates and fat by the liver and muscles. This is in line with our results which show a lower fat oxidation after high GI mixed meals. Notably, over 10 h, low GI mixed meals resulted in an increased oxidation of 4.7 g of fat which translate to around 42 kcals burned from fat, which could accumulate to around 300 kcals per week. It must be noted that the increased energy expenditure is lower when decreased carbohydrate oxidation is taken into account. Hall et al. have shown that it may take only 7.5 kcal extra per day to explain the current epidemic of obesity [48]. This may indicate that even in a sedentary state, a low GI diet based on locally available foods, not only lowers blood glucose but also enhances fat oxidation and may play a key role in weight regulation and maintenance.

Investigating glycaemic index and glycaemic response of locally consumed foods revealed that as expected, all high GI meals resulted fast absorption and a higher glucose response, the highest postprandial glucose response was observed after the high GI breakfast. Poon kueh is steamed glutinous rice and is commonly consumed as a breakfast amongst Asians. The mean areas under the postprandial tissue glucose curve after ingestion of the high GI breakfast was two-fold higher compared to the low GI breakfast. The high GI dinner meal, which consisted of boiled glutinous rice and side dishes, also exhibited a much higher glucose response compared to the low GI dinner. Glutinous rice has been reported for its high starch digestibility that causes sharp spikes in blood glucose responses [49].

No serum insulin measurements were available in this study which could further enhance the link between glycaemic response and fuel utilization. Protein oxidation had to be assumed to be constant as it could not be measured in a time-specific way and was based on the total 10 h cycle in the WBC [50]. Another limitation was the inclusion of only male subjects to avoid metabolic variability in insulin sensitivity and glycaemic response in females due to the menstrual cycle, which might bias the results due to the gender of individuals [51]. An important feature of the current study was the use of commonly consumed Asian mixed meals. Not all micro-nutrients and the sources of proteins and fats were possible to match and this can have an effect on glycaemic response, however percentage calories from carbohydrates, fat and protein were matched for the low and high GI diet as this is the most important in meal composition.

## Conclusions

Novel findings of this study are the ability to reduce glycaemic response over 24 h and increase fat oxidation of a 24 h low GI mixed meal plan. Unique to our study was the use of Asian and commonly consumed low GI foods to construct a low GI diet and the simultaneous measurement of blood glucose (continuous glucose monitoring) and substrate oxidation using a whole body calorimeter. The improvement of glycaemic response and decrease in variability found in sedentary, normal weight Asians with local low GI foods are important because the Asian phenotype has been shown to be more susceptible to Diabetes compared to Caucasians [8, 9]. Additionally in Asia, people predominantly live on a high glycaemic, high carbohydrate diet and are thus susceptible to weight gain and obesity similar to observations from animal studies [37]. Future research has to be conducted to elucidate on the link between blood glucose levels and fat oxidation shown in this study, in order to quantitate how insulin and other hormones may play a role in tissue accretion and substrate oxidation. Our observations provide public health support for the encouragement of healthier nutrition choices by consuming low GI foods.

## Abbreviations

BMI: Body mass index
BMR: Basal metabolic rate
CGM: Continuous glucose monitoring
CHO: Carbohydrate
GI: Glycaemic index
iAUC: Incremental area under the curve
MAGE: Mean amplitude of glycaemic excursion
RQ: Respiratory quotient
WBC: Whole body calorimeter
